# Prävention und Kostenkontrolle im Gesundheitswesen: Routinedatenanalyse und effektives Gesundheitsmarketing als Vorteile im Krankenkassenwettbewerb?

**DOI:** 10.1007/s11553-023-01021-y

**Published:** 2023-03-17

**Authors:** Tobias Effertz

**Affiliations:** Fakultät für Betriebswirtschaft, Institut für Recht der Wirtschaft, Universität Hamburg, Moorweidenstr. 18, 20148 Hamburg, Deutschland

**Keywords:** Big Data, Prädiktionsmodell, Machine Learning, Real World Data, Ungesunde Lebensstile, Bid data, Prediction model, Machine Learning), Real world data, Unhealthy lifestyles

## Abstract

**Hintergrund:**

Das deutsche Gesundheitswesen hat mit hohen Kosten zu kämpfen. Neben den aktuellen finanziellen Belastungen im Zusammenhang mit der Coronapandemie verfolgt die deutsche Bevölkerung mehrheitlich einen ungesunden Lebensstil der zukünftig zu weiteren Neuerkrankungen und assoziierten Kosten führen wird.

**Ziel der Arbeit:**

Dieser Beitrag skizziert, wie mit Hilfe von Routinedatenanalysen der Gesetzlichen Krankenversicherung (GKV) Ansatzpunkte für Maßnahmen auf allen Ebenen der Prävention entwickelt werden können, die effektiv Krankheitsentstehungen verhindern, -progresse reduzieren und die Fähigkeit zur Beitragszahlung absichern können. Diese sollen durch effektives Gesundheitsmarketing Versicherte zu gesundheitsverbessernder Leistungsinanspruchnahme animieren.

**Material und Methode:**

Es wird eine Idee entwickelt und diskutiert, in der ergänzend zu den bestehenden Möglichkeiten der GKV auf präventives Gesundheitsverhalten hinzuwirken, Ergebnisse der Sekundärdatenanalyse für Präventionsmaßnahmen und -verhalten nutzbar gemacht werden können.

**Ergebnisse und Schlussfolgerung:**

Eine Machine-learning-gestützte Analyse bildet den Kern einer Klasse von Prädiktionsmodellen für die Prävention von Krankheiten. Diese Modelle setzen an unterschiedlichen Patientenmerkmalen in den Routinedaten der GKV an und liefern Empfehlungen für Präventionsmaßnahmen, die für eine zielgerichtete und kosteneffektive Ansprache beispielsweise mittels mHealth genutzt werden können. Die hohen Einsparungspotenziale im Gesundheitswesen sowie die Möglichkeiten der Gesetzlichen Krankenkassen diese datenanalytisch gestützt zu erschließen eröffnen eine sinnvolle Perspektive zu nachhaltiger Kostenkontrolle.

## Abstract (en)

**Background:**

The German healthcare system is struggling with increasing costs. Besides the current extra burden due to the corona pandemic, the vast majority of Germans pursue unhealthy lifestyles which will lead to additional morbidity and costs in the future.

**Objectives:**

This contribution sketches out an idea, on how analyses on claims data from the Statutory Health Insurance (SHI) in Germany may contribute to better usage of preventive health services to counteract the onset and progress of morbidities and hence ensure stable premium income from the insured. Effective health communication may further enable demand for preventive measures.

**Materials and methods:**

An idea is developed and discussed in which, in addition to the existing possibilities of the SHI to work towards preventive health behavior, results of secondary data analysis may be used for preventive measures and behavior.

**Results and conclusions:**

A machine-learning-based analysis is the core of a class of prediction models for prevention of illnesses. The models exploit the information from routine data and provide recommendations for prevention services, which in turn may be promoted to the insured via targeted, tailored, and personalized communication, e.g., via mHealth apps. The high potential for cost reductions as well as the possibilities to exploit them via data analytics provide a promising perspective for sustained cost control in the healthcare sector.

Die Gesetzliche Krankenversicherung (GKV) hat mit steigenden Kosten zu kämpfen. Nicht nur die Coronapandemie, sondern auch der große Anteil ungesunder Lebensstile in der deutschen Bevölkerung führt zu nachhaltigen finanziellen Belastungen. Es müssen neue Wege gefunden werden, um die künftige Kostensituation im Gesundheitswesen kontrollierbar zu halten. Als wesentliche Alternative zu Korrekturen auf der Einnahmenseite sind wirkungsvolle Präventionsmaßnahmen der Schlüssel zur Kostenstabilisierung bei gleichzeitig besserer Bevölkerungsgesundheit. Für Krankenkassen stellt sich die Frage, wie sie ihre Versicherten tatsächlich auch zu einer optimierten Inanspruchnahme von Präventionsleistungen motivieren können.

## Ausgangslage

Das Problem von Finanzierungsdefiziten aufgrund steigender Kosten im deutschen Gesundheitswesen ist von wiederkehrender Aktualität geprägt. Momentane Hilfsmaßnahmen setzen an der Einnahmenseite an und blenden dadurch einen zentralen Aspekt aus: die Kostenreduktion im Gesundheitswesen durch effektive Präventionsmaßnahmen.

Ungesunde Lebensstile wie Rauchen, Adipositas und übermäßiger Alkoholkonsum finden sich in großen Teilen der deutschen Bevölkerung [1, 2]. Etwa 25 % der Bevölkerung sind krankhaft übergewichtig, etwa 27 % rauchen und zwischen 3 und 4 Mio. Menschen trinken gesundheitsschädliche Mengen an Alkohol. In der Konsequenz führen diese ungesunden Lebensstile zu erhöhtem Krankheitsleid und höheren Kosten im Gesundheitswesen. Um eine Reduktion der Krankheitslast und damit der Kosten im Gesundheitssystem herbeizuführen, sind wirksame Präventionsmaßnahmen, die vor Einsetzen einer Krankheit aber auch im Krankheitsverlauf weitere Verschlechterungen der Gesundheit verhindern, zentral. Damit kommt dem individuellen, gesundheitsförderlichen und präventivem Verhalten eine zentrale Rolle zu, die nicht nur das Individuum selbst, sondern auch Mitmenschen vor Krankheiten und der Exposition mit gesundheitlichen Risikofaktoren wie Krankheitserregern oder Tabakrauch schützt.

Ein sich neu eröffnender Ausweg, um Informationen aus der Domäne der Real World Data (RWD) für effektive Präventionsmaßnahmen zu nutzen, sind GKV-Routinedaten [3]. Diese fallen routinemäßig bei Abrechnungs- und Überweisungsvorgängen im GKV-System an und beinhalten neben Informationen zu Diagnosen und durchgeführten medizinischen Maßnahmen bei Patienten auch sozioökonomische Merkmale wie Ausbildungsniveau, Berufsgruppe, Familienstatus und eingeschränkt auch den Migrationshintergrund [1, 4]. Die Analyse von Routinedaten kann der Ausgangspunkt einer Präventionsstrategie zu Kostensenkung der Krankenkassen bilden.

### Kostensenkungspotenziale durch effektive Prävention

Aufgrund der geschilderten gesundheitsschädlichen Konsummuster drohen weitere Steigerungen der Kosten in den kommenden Jahrzehnten. Aktuelle Kostenschätzungen zeigen signifikante Reduktionspotenziale, die durch wirksame Präventionsmaßnahmen, etwa kosteneffektive Frühinterventionen, Primärprävention aber auch Rehabilitationsmaßnahmen, realisiert werden könnten [1, 2, 5]. Mit dem Präventionsgesetz 2015 wurde eine gesetzliche Grundlage für die GKV geschaffen, ihren Patienten verhaltenspräventive Maßnahmen mit einem wenn auch geringen Kostensatz zu finanzieren. Da verhaltenspräventive Maßnahmen als wenig effektiv gelten, müssen diese hinreichend günstig sein, um eine Kosteneffektivität sicherzustellen. Damit fällt der Informations- und Analysetechnik, die aufzeigt, in welcher Situation ein Patient auf relevante Präventionsmaßnahmen aufmerksam gemacht werden soll, eine wichtige Rolle zu.

### Real World Data, Big Data und Routinedaten im Gesundheitswesen

Mit RWD sind in der Literatur bislang Daten gemeint, die im Gesundheitswesen aus teils von Menschen erstellten, teils automatisierten Datengenerierungen meist „routinemäßig“ im Rahmen von IT-Prozessen entstehen [6]. Man nennt solche Daten auch Sekundär- oder Routinedaten.

Aus den RWD lassen sich mit statistischen Methoden Befunde – Real World Evidenz (RWE) – ableiten, die für die Entscheidungsfindung verschiedener Fragestellungen im Gesundheitswesen relevant sein können. Bei der Ergründung und Nachweisführung von Kausalitäten einzelner therapeutischer Maßnahmen zur Gesundheitsverbesserung hat man bislang in der Medizin mit Blick auf den Goldstandard der randomisiert kontrollierten Studien („randomized controlled trial“, RCT) den RWD eine hinreichende Geeignetheit abgesprochen, auch wenn andere Wissenschaftsdisziplinen wie die Ökonomie über ein statistisches Instrumentarium für kausale Interpretationen von Befunden verfügen [7]. Mittlerweile akzeptieren aber auch Medizin und Epidemiologie zunehmend Routinedaten als zusätzliche Informationsquellen für Entscheidungsfindungen im Gesundheitswesen. Die Routinedaten der GKV qualifizieren sich aufgrund der großen Versichertenbestände einiger Krankenkassen als „Big Data“, d. h. anhand der drei „V“, die für die englischen Begriffe „volume“ (große Datenmengen), „variety“ (hohe Datenvielfalt) und zunehmend auch „velocity“ (hohe Geschwindigkeit der Datengenerierung und -auswertung) stehen [8]. GKV-Routinedaten werden im Folgenden bezüglich möglicher Ansatzpunkte für Präventionsmaßnahmen diskutiert.

### Präventionskonzepte und vulnerable Gruppen

Präventionsmaßnahmen lassen sich nach dem zeitlichen Ansatzpunkt in Bezug auf zu verhindernde oder zu verbessernde Krankheiten unterscheiden [9]: Maßnahmen der Primärprävention setzen dabei vor dem Auftreten einer Krankheit an und stellen ein Risikofaktormanagement dar. Beispiele sind etwa Aufklärungskampagnen gegen das Rauchen und schädlichen Alkoholgenuss oder Impfungen gegen das neue Coronavirus SARS-CoV‑2 („severe acute respiratory syndrome coronavirus 2“). Sekundärprävention versucht Krankheiten in einem häufig noch symptomlosen Frühstadium zu erkennen und durch sich anschließende Frühinterventionen zurückzuführen oder einzudämmen. Maßnahmen der Tertiärprävention schließlich versuchen die Situation von Personen mit chronischen Krankheiten zu verbessern, indem sie in Form der Rehabilitation versuchen, eine soziale Teilhabe zu ermöglichen oder Krankheitsprogresse zu verlangsamen.

Der Begriff der „vulnerablen Gruppe“ ist zentral für Präventionskonzepte. Er definiert eine Personengruppe mit bestimmten gemeinsamen Eigenschaften oder Merkmalen, die im Vergleich zum Rest der Gesellschaft aufgrund ihrer Eigenschaften nicht adäquat auf gesundheitliche Herausforderungen reagieren kann. Dabei ist entscheidend, dass die Klassifizierung als „vulnerabel“ kontextabhängig ist [10]. So können z. B. auch gesunde Gruppen vulnerabel sein, wenn etwa Sprachbarrieren in Impfkampagnen oder religiöse Ansichten eine adäquate medizinische Versorgung verhindern.

## Ansatzpunkte für Präventionsmaßnahmen in GKV‑Routinedaten

**Abb. 1:**
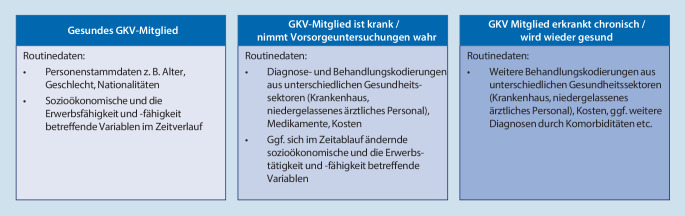
Datengenerierung über Versicherte im System der Gesetzlichen Krankenversicherung (GKV)

Grundsätzlich existieren drei große Merkmalsschwerpunkte in GKV-Routinedaten, an denen Präventionsmaßnahmen ansetzen können: 1. Daten, die Informationen über Demografie und sozioökonomischen Status der Versicherten enthalten (demografische Daten), 2. Daten über Kosten und in Anspruch genommene Leistungen im Gesundheitssektor (Leistungsdaten) sowie 3. Diagnosedaten, die in unterschiedlichen Sektoren des Gesundheitssystems entstehen (Krankheitsdaten). Diese sind für sich genommen bereits für einige Fragestellungen im Bereich der Prävention geeignet, entfalten aber v. a. in Kombination über die verschiedenen Gesundheitssektoren hinweg eine hohe Aussagekraft. Abb. 1 zeigt auf, welche wesentlichen Routinedaten eines Patienten im gesunden Zustand und bei Krankheit vorliegen:

### Sozioökonomische und demografische Variablen

Die Datenmerkmale zu demografischen und sozioökonomischen Eigenschaften bieten zahlreiche Ansatzpunkte für Prävention: Eine Reihe von primär- und sekundärpräventiven Maßnahmen setzen explizit am Alter eines Adressaten an. So fokussieren beispielsweise Informationskampagnen der Bundeszentrale für gesundheitliche Aufklärung für die Prävention sexuell übertragbarer Erkrankungen oder gegen exzessiven Alkoholkonsum insbesondere junge Zielgruppen. Es gibt geschlechtsspezifische oder den Migrationshintergrund berücksichtigende Kampagnen. Informationsmaterialen zur Prävention von Arbeitsunfällen existieren branchenspezifisch und es lassen sich aus dem erfassten Merkmal der Erwerbsunfähigkeit bzw. des Pflegestatus Ansatzpunkte für tertiärpräventive Maßnahmen, etwa zur Erhaltung und Verbesserung von Mobilität und Selbstständigkeit, ableiten.

Die Früherkennungsuntersuchungen von Krankheiten, neben der Frühintervention eine der beiden konstituierenden Säulen der Sekundärprävention, wird üblicherweise ebenfalls in Abhängigkeit des Alters in regelmäßigen Intervallen empfohlen^1^. Einen entscheidenden Beitrag zur Identifizierung und Segmentierung von vulnerablen Gruppen bildet die Altersvariable auch in Kombination mit anderen Variablen. So können das Herkunftsland, die Stellung im Beruf sowie der erreichte Ausbildungsgrad wichtige Indikatoren sein, eine vulnerable Gruppe schneller zu identifizieren, mit relevanten Informationen zu adressieren und ggf. an Termine zur Früherkennung zu erinnern. Beispielsweise weisen Arbeiter der chemischen Industrie oder im Bergbau ein erhöhtes Lungenkrebsrisiko aufgrund der beruflichen Tätigkeit auf. Tab. 1 greift beispielhaft unterschiedliche Merkmalskonstellationen in den Sekundärdaten mit den jeweiligen einschlägigen vulnerablen Gruppen und möglichen abgeleiteten Präventionsmaßnahmen auf.

**Tab. 1:** Demografische und sozioökonomische Datenmerkmale, korrespondierende vulnerable Gruppen und ableitbare Präventionsmaßnahmen

Präventionsmaßnahme	Präventionsebene	Vulnerable Gruppe	Merkmal in GKV-Routinedaten	Merkmalsausprägung
Push-Nachricht auf das elterliche Smartphone zur Erinnerung an U‑Untersuchungen (Kind)	Primärprävention	Kinder	Alter familienversicherter Kinder	Alter in Jahren
Infobroschüre zu sexuell übertragbaren Krankheiten und deren Prävention	Primärprävention	Jugendliche und junge Erwachsene	Alter Versicherter/Familienversicherter	Alter in Jahren
Hinweis per Push-Mitteilung auf versäumte Früherkennungsuntersuchungen für Männer und Frauen (z. B. Vorsorge Mamma‑, Prostatakarzinom)	Sekundärprävention	Erwachsene ab Mitte 40	Alter Versicherter/Familienversicherter und Geschlecht Versicherter/Familienversicherte	Alter in Jahren/Geschlecht männlich/weiblich
Anpassung der Sprache in mHealth-Gesundheitsinformationen	Primär-, sekundär-, tertiärpräventiv	Personen mit Migrationshintergrund	Staatsangehörigkeit	Deutschland/andere Staaten
„Reha vor Pflege“ – Informationsbroschüren über Maßnahmen zur Förderung und Erhaltung der Funktionalität und Selbstständigkeit bei Pflegestatus	Tertiärpräventiv	Pflegebedürftige Menschen	Pflegestatus	Pflegestufen und Pflegegrade

*GKV* Gesetzliche Krankenversicherung

Interessant aus Sicht der GKV an dieser Herangehensweise ist, dass die Routinedatenanalysen und Informationszeitpunkte, an denen Versicherte von ihrer Krankenkasse auf Prävention aufmerksam gemacht werden, unabhängig von medizinischen Leitlinien und dem heute üblichen Erinnerungsmanagement niedergelassener Ärzte erfolgen können. Die Gesetzlichen Krankenkassen verfügen damit über eigene Analysemöglichkeiten zur Verbesserung der Gesundheitszustände ihrer Versicherten und können ergänzend zu den Empfehlungen der Leistungserbringer eigene Möglichkeiten finden an vulnerable Personen in ihrem Versichertenkollektiv heranzutreten. Dies ist ihnen im Rahmen des SGB V explizit gestattet.

### Diagnosedaten

Bei den Gesetzlichen Krankenkassen laufen von Ärzten kodierte und übermittelte Diagnosedaten aus sämtlichen Gesundheitssektoren über einen Patienten in einer Datenbank zusammen. Dies hat den Vorteil, dass sich Fehlkodierungen im Zeitablauf nivellieren [1] und ungenaue oder falsche Diagnosen durch Zweitmeinungen und aus anderen medizinischen Zusammenhängen heraus neu bewertet werden können. Dies zeigt sich am Beispiel des schädlichen Alkoholkonsums, der z. B. durch den Hausarzt aufgrund persönlicher Einstellungen als nicht relevant und üblich angesehen werden mag, während ein Klinikarzt im akuten Fall einer Alkoholvergiftung infolge Rauschtrinkens den entsprechenden Diagnoseschlüssel meist richtig kodieren wird [5]. Auch Betriebsärzte könnten zum Schutz des Arbeitnehmers vor möglichen Haftungsrisiken auf eine korrekte Kodierung verzichten, da in Deutschland der Unfallversicherungsschutz eines alkoholisierten Arbeitnehmers am Arbeitsplatz erlischt und Ärzte aus diesem Grund von einer Feststellung von Trunkenheit am Arbeitsplatz absehen könnten [11]. An diesem Beispiel zeigt sich der Vorteil der Informationsverdichtung in den GKV-Routinedaten, die problematischen Alkoholkonsum systematischer und transparenter abbilden, als dies eine Momentaufnahme eines einzelnen Leistungserbringers vermag.

Diagnosedaten sind, auch wenn diese als nicht gesichert kodiert werden, Anhaltspunkte für Symptome und Krankheiten und damit für präventive Frühinterventionen bei erstmaligem Auftreten sowie Tertiärprävention bei andauernder Diagnosestellung.

Die bereits erwähnte Möglichkeit der GKV, eigene Analysen durchzuführen und aus diesen Empfehlungen für Präventionsmaßnahmen abzuleiten, lässt sich grundsätzlich auch für den Bereich der Diagnosedaten insbesondere für prognostische Zwecke von Krankheitsentstehungen und -progressen nutzen. So ließen sich Fallgruppen von Datenmerkmalskonstellationen im Vorfeld manifester chronischer Erkrankungen identifizieren, die entweder korrelativ vulnerable Gruppen für gezieltere sekundärpräventive Maßnahmenansprachen ermitteln oder im besten Fall sogar eine kausale Interpretation erlauben. In beiden Fällen könnte ein „Frühwarnsystem“ auf Basis von GKV-Routinedatenanalysen eine entsprechende Empfehlung zur Untersuchung an Versicherte versenden, wenn sich bestimmte Merkmalsausprägungen verdichten.

### Daten über Leistungsinanspruchnahmen und Kosten

Der dritte große Datenbereich, aus welchem Präventionsmaßnahmen abgeleitet werden können, sind die Erfassung von Leistungen im Gesundheitssystem und deren Kosten. Interessant ist hierbei die hohe inferentielle Aussagekraft, die Daten über Leistungsinanspruchnahmen und insbesondere Arzneimittelverschreibung für das Vorliegen bestimmter Krankheiten haben, wie ein jüngerer Bereich der Sekundärdatenanalyse aufzeigt [12, 13]. Dies ist insbesondere hilfreich für Gesundheitssysteme, in welchen Diagnosedaten routinemäßig nicht erhoben werden oder nicht verfügbar sind.

Damit sind beide Leistungs- und Diagnosedaten aber nicht redundant, sondern ergänzen sich sinnvoll: ein Diabetes mellitus Typ 1 mit hohen Behandlungskosten kann beispielsweise auf das Vorliegen weiterer unentdeckter Komorbiditäten oder eine schlecht eingestellte Pharmakotherapie hindeuten. Hohe Kosten über einen längeren Zeitraum mit wechselnden Diagnosen mögen darauf hindeuten, dass die Grunderkrankung noch nicht richtig erkannt wurde und im Zusammenhang mit schweren chronischen Erkrankungen können hohe Kosten auch den Beginn der „End-of-life“-Phase nahelegen. Tab. 2 zeigt exemplarisch auf, welche Möglichkeiten zur Prävention sich aus der speziellen Diagnosekodierung und Leistungsinanspruchnahme in Kombination mit sozioökonomischen und demografischen Daten ergeben können.

**Tab. 2:** Beispiele konkreter Ansatzpunkte für Prävention mit Diagnosedaten

Präventionsmaßnahme	Vulnerable Gruppen	Präventionsebene	Merkmale in GKV-Routinedaten	Auslösendes Merkmal
Hinweis an Patienten, dass kritische Krankheitskonstellation vorliegt, die Gesundheitszustand potenziell verschlechtern und die der ärztlichen Abklärung bedürfen	Vorerkrankte mit bestimmten Diagnosekonstellationen und -verläufen	Sekundär‑/Tertiärpräventiv	Diagnosekodes im Zeitablauf	Überschreiten kritischer Häufigkeiten, Zusammenfallen bestimmter Diagnosekombinationen
Empfehlung zu Arztgespräch nach wiederholter Alkoholentgiftung im Krankenhaus „Screening & Brief Intervention“	Jugendliche und Erwachsene	Primär- und Sekundärpräventiv	Häufigkeit von Diagnosekode zur Alkoholintoxikation/Altersvariable	Frühes wiederholtes Auftreten von Alkohointoxikation in jungen Jahren
Push-Mitteilung zur Arztkonsultation nach spätem Erkennen eines Schwangerschaftsdiabetes zwecks Vermeidung der Chronifizierung	Schwangere Frauen/Frauen die gerade entbunden haben	Sekundär‑/Tertiärpräventiv	Diagnosekodes gesicherte Schwangerschaft und Zustand nach Schwangerschaft Diagnosekode Diabetes Typ 4	Zusammenfallen von Schwangerschafts- und Diabetesdiagnose
Hinweis per Push-Mitteilung über verbesserten Umgang mit chronischen Infektionen wie verschleppter Borreliose	Borrelioseinfizierte	Tertiärpräventiv	Diagnosekode Borrelioseinfektion	Wiederholtes Auftreten des Borreliosekodes über vier Quartale
Informationsbroschüren zur verbesserten Wundheilung und Nachsorge nach chirurgischer Tumorentfernung	Patienten in Krebstherapie	Tertiärpräventiv	Diagnosekodes und Operationsschlüssel	OPS-Kode nach Diagnosestellung

*GKV* Gesetzliche Krankenversicherung, *OPS* Operationen- und Prozedurenschlüssel

## Machine-learning-gestützte Prädiktionsmodelle zur Präventionsmaßnahmenoptimierung

Das Vorliegen der geschilderten Datenmerkmale erlaubt auch eine Modellierung von Krankheits‑, Krankheitskosten- und Behandlungsverläufen von Versicherten im Rahmen sog. „Prädiktionsmodelle“ [14]. Mit diesen lassen sich idealerweise Zustände im Verlauf einzelner Versicherter identifizieren, die die Empfehlungsmitteilung für Präventionsmaßnahmen seitens der Krankenkasse auslösen können. Da Kostenreduktionen aus wirtschaftlichen Gründen und Verbesserungen des Gesundheitszustands durch Prävention langfristig keine konfliktären Ziele für die Krankenkassen und im Sinne der Versicherten sind, da dies einen niedrigeren Zusatzbeitrag und bessere Gesundheit bedeutet, können bessere künstliche Intelligenz (KI)-gestützte Prädiktionsmodelle für Präventionsmaßnahmen ein essentieller Erfolgsfaktor im Wettbewerb der Krankenkassen sein.

**Abb. 2:**
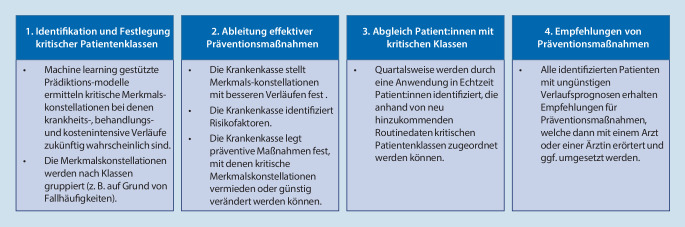
Veranschaulichung des logisch-analytischen Ansatzes zur Identifizierung vulnerabler Gruppen und Auslösezeitpunkte präventivmedizinischen Handelns

Prädiktionsmodelle werden aktuell beispielsweise für die Abschätzung des kurzfristigen Mortalitätsrisiko von COVID-19-Patienten („coronavirus disease 2019“; [15]) oder zur verbesserten Wirbelsäulenchirurgie [16] genutzt. Auf Basis von GKV-Routinedaten ließen sich ebenfalls Prädiktionsmodelle aufstellen. Nachstehende Abb. 2 veranschaulicht beispielhaft abstrakt, wie ein automatisierter Prozess für die Steuerung von Präventionsempfehlungen grundsätzlich aussehen könnte:

Für die Prädiktionsmodelle haben sich im Rahmen der Machine-learning-Thematik zwei Modellklassen herausgebildet die für die Identifizierungsaufgabe, kritische Gruppen für Präventionsmaßnahmen ausfindig zu machen, geeignet erscheinen: Zum einen ein strukturentdeckendes Verfahren z. B. eine Cluster- oder latente Klassenanalyse, das auf Basis der Informationen über Soziodemografika, Diagnosen und Leistungsdaten verschiedene kritische Klassen identifiziert, bei denen spezifische Präventionsmaßnahmen vor dem Hintergrund der Kostenreduktionen und Gesundheitsverbesserungen angezeigt erscheinen. Sobald ein Versicherter aufgrund seiner übermittelten Merkmalskonstellationen in den Routinedaten in eine solche Klasse fällt, wird vom IT-System ein Hinweis erstellt, der dann zu einer Informations- oder Werbemaßnahme für eine passende Präventionsmaßnahme führt.

Einem zweiten Modelltypus entspricht im Machine-learning-Kontext die Lasso-Regression, bei der die in die Regression aufzunehmenden erklärenden Variablen durch Abgleich mit Datenanpassungsstatistiken und Vorhersagegenauigkeit zur Erklärung eines Gesundheitszustands durch ein Analyseprogramm selbst bestimmt werden. Beide Modelltypen lassen sich über „lernende“ Prozeduren wie „Support Vector Machines“ oder „Random Forests“ implementieren, die eine Aufteilung in präventionsrelevante und nicht relevante Zustände in Abhängigkeit der Merkmalshäufungen in der Grundgesamtheit vornehmen. Die prognostische Güte beider Modelltypen hängt von Speicherkapazitäten und Rechenleistungen des zugrundeliegenden IT-Systems ab und es empfiehlt sich, dem Gebot der statistischen Sparsamkeit folgend, bei der Modellkonzeption zunächst auf die Sachverhalte zu fokussieren, die aus Gesundheits- und Kostensicht besonders drängend sind, und das System dann langsam zu vervollständigen.

## Erfolgreiche Maßnahmenimplementierung durch effektives Gesundheitsmarketing

Über die gewonnenen Erkenntnisse in Form abgeleiteter sinnvoller Präventionsmaßnahmen und -zeitpunkte aus der Sekundärdatenanalyse sollten die Datenhalter, also die Gesetzlichen Krankenkassen, die Versicherten durch wirksame Ansprache direkt informieren. Dies ist der entscheidende konzeptionelle Unterschied zu den bisherigen Möglichkeiten der Krankenkassen, ihre Versicherten im Rahmen von Bonusprogrammen finanzielle Anreize zu bieten, Präventionsmaßnahmen wie Früherkennungsuntersuchungen wahrzunehmen. Bisher kam der Handlungsimpuls vom Versicherten, während bei einer Ansprache im Sinne eines Werbe-„Cues“ oder „Hinweisreizes“ in Kombination mit einem finanziellen Anreiz durch die Krankenkassen, der Versicherte reagiert. Auf Basis der Befunde zur affektiven und impulsiven Cue-Reaktivität im Marketing [17] und finanzieller Anreize erscheint die direkte Ansprache durch die Krankenkasse Erfolg versprechender, als darauf zu warten, dass ein Versicherter regelmäßig zur Vorsorgeuntersuchung geht.

Die Pflicht der Gesetzlichen Krankenkassen, ihre Patienten hinsichtlich der Erhaltung ihrer Gesundheit zu beraten und damit aktiv Präventionsmaßnahmen vorzuschlagen und auf Angebote hinzuweisen, ergibt sich bereits aus dem § 1 des Sozialgesetzbuches V (SGB V)^2^. Laut dem Präventionsgesetz von 2015 müssen die Gesetzlichen Krankenkassen jährlich einen bestimmten Geldbetrag pro Versicherten für Präventionsleistungen ausgeben. Dabei erscheint angesichts der knappen Budgetierung für Prävention insbesondere die Empfehlung von Maßnahmen idealerweise über mHealth-Anwendungen, also Anwendungen, die als elektronische Information per E‑Mail oder App auf das Smartphone geschickt werden können, als kosteneffektiv, da diese mit hinreichend persönlichem Zuschnitt in der Ansprache automatisiert zugestellt werden kann, wenn z. B. ein Versicherter ein bestimmtes Alter für eine Vorsorgeuntersuchung erreicht hat.

Insofern es sich bei der Beratung der Krankenkassen um eine rein informative Mitteilung an die Versicherten (z. B. über Zeitpunkte zur Früherkennungsuntersuchung) handelt, sind diese, solange der Versicherte dem nicht ausdrücklich widerspricht, direkt kommunizierbar. Meist hat aber eine rein informative Darstellung von Hinweisen auf Versorgungs- und damit auch Präventionsmaßnahmen mit steigender Komplexität kaum einen Effekt im Antwortverhalten, d. h. die Informationsvermittlung muss im Rahmen einer wirksamen Gesundheitskommunikation erfolgen.

Den Gesetzlichen Krankenkassen sind bislang durch neue gesetzliche Regelungen und Rechtsprechung beim Marketing insbesondere der (An‑)Werbung von Mitgliedern Grenzen gesetzt: So ist es beispielsweise nach der Krankenkassen-Werbemaßnahmen-Verordnung (KKWerbeV) nicht erlaubt, gezielt Risikoselektion bei der Anwerbung von neuen Mitgliedern zu betreiben und bespielweise gezielte Auswahl- und Akquiseprozesse zur Verbesserung der Risikostruktur in den Versichertenkollektiven durch Fokussierung auf hohe Einkommensgruppen durchzuführen. Auch ist es mit Urteil des Bundessozialgerichts vom 08.10.2019 (Aktenzeichen B 1 A 3/19 R) nicht erlaubt, in Konkurrenz zu zugelassenen Leistungserbringern wie Ärzten und Krankenhäusern eigene Leistungen des Versorgungsmanagements zu unterbreiten^3^.

Allerdings dürfen (und sollen) nach der die Werbung konkretisierenden KKWerbeV die Gesetzlichen Krankenkassen ihren Mitgliedern Präventionsangebote machen, auch wenn sich diese an spezielle Teilgruppen wie adipöse Personen oder Raucher richten. Dies darf auch mithilfe emotionaler Werbung geschehen und die Maßnahmen können mit Bonusprogrammen der Krankenkassen kombiniert werden, in welchen finanzielle und nichtfinanzielle Anreize zu gesundheitsförderndem Verhalten gesetzt werden. Nach § 65a SGB V sind die Gesetzlichen Krankenkassen in der Gestaltung ihrer Bonusprogramme für Präventionsmaßnahmen frei und können diese per Satzung selber regeln. Man erkennt wie wichtig es ist, die abgeleiteten Präventionsmaßnahmen effektiv zu vermarkten und dabei auch mit finanziellen Anreizen zu arbeiten, sofern die Gesetzlichen Krankenkassen eine signifikante Kostenreduktion erreichen wollen. Bislang häufig genutzte Informationskampagnen für Vorsorgeuntersuchungen gelten dem gegenüber als wenig effektiv [18].

## Diskussion und Limitationen

Der in diesem Beitrag skizzierte Vorschlag sieht vor, dass Routinedaten der Gesetzlichen Krankenkassen zur Analyse von Gesundheitsentwicklungen der Versicherten genutzt und bei sich abzeichnenden riskanten oder sich verschlechternden Gesundheitsverläufen von der Krankenkasse Empfehlungen zur Förderung der Gesundheit und Prävention von Krankheit an die Patienten ergehen. Dabei sollen idealerweise mittels mHealth-Anwendungen von der Krankenkasse versandte Push-Nachrichten direkt auf dem Smart-Phone der Patienten zu einem ärztlichen Beratungsgespräch führen. Dies bedeutet eine größtenteils bewusst herbeigeführte Redundanz an Gesundheitsinformationen für den Patienten: So kann potenziell ein behandelnder Arzt oder jeder andere Leistungserbringer über die Prävention bestimmter Risiken wie Rauchen oder schädlichen Alkoholkonsum aufklären und zu entsprechenden Maßnahmen raten. Es ist aber gerade die Redundanz und die Ansprache seitens der Krankenkassen, die – ggf. gepaart mit finanziellen Anreizen oder Bonusprogrammen – eine zusätzliche und wirksame Ansprache an die Patienten vornehmen kann. Damit geht dieser Ansatz über die elektronische Patientenakte hinaus, welche lediglich vorsieht, dass Ärzte Informationen auf Initiative des Patienten digital bereitstellen.

Der Vorschlag, dass Krankenkassen ihre Versicherten aktiv zu bestimmten medizinischen Maßnahmen anregen, wird insbesondere bei Medizinern auf Kritik stoßen, die eine schädliche Überversorgung an Früherkennungsmaßnahmen mit zweifelhaftem Nutzen für die Patienten befürchten sowie den Leistungserbringern im Gesundheitswesen eine Überaktivität attestieren, die in vielen Fällen nicht als medizinisch sinnvoll gilt [19]. Gleichwohl muss man konstatieren, dass die Teilnahmeraten an verschiedenen etablierten Früherkennungsscreenings deutlich unter 50 % liegen [20–22]. Das hohe Niveau von Adipositas und ungesunden Lebensstilen in Deutschland trägt maßgeblich zu einer Reihe schwerer Erkrankungen bei, durch welche die Kostensituation der GKV angespannt bleibt. Damit hat eine Krankenkasse v. a. Anreize, durch sinnvolle Präventionsempfehlungen nachhaltig zukünftige Kostenrisiken zu reduzieren und damit gleichzeitig die Gesundheit des Patienten zu erhalten. Gemäß dem medizinischen Grundsatz des „Primum non nocere“ bleibt es dabei entscheidend, dass die Krankenkassenempfehlung nicht parallel oder sogar gegen einen ärztlichen Rat erfolgt, sondern idealerweise zu einem Arztkontakt führen soll, bei welchem die Empfehlung abgeklärt und damit das Arzt-Patienten-Verhältnis verbessert wird.

Jede einzelne Krankenkasse wird aus wirtschaftlichem Kalkül überlegen, welche Präventionsempfehlungen für ihren Versichertenpool sinnvoll sind. Die Maßnahmenbündel können zwischen den Versichertenkollektiven einzelner Krankenkassen sehr unterschiedlich ausfallen, je nachdem, wie prävalent einzelne Krankheitsausprägungen oder Soziodemografika ausfallen. Da der Versicherte über die Charakteristika des Versichertenpools seiner Krankenkasse nicht informiert ist, könnte es damit zu ausbleibenden Empfehlungen für einzelne Versicherte kommen, obwohl diese aus individuell gesundheitlicher Sicht sinnvoll wären. Dem könnten Versicherte letztlich nur mit einem Krankenkassenwechsel entgegenwirken.

Mit dem Brustkrebsscreeningprogramm für Frauen zwischen 50 und 65 besteht bereits eine ähnliche Struktur; hier erfolgt ein Anschreiben zum jährlichen Screening auf Basis von Daten der Einwohnermeldeämter. Da bei allen Screeninguntersuchungen ein falsch-positiver Befund mit psychischen Belastungen und im schlimmsten Fall unnötigen Eingriffen verbunden sein kann, muss geklärt werden, ob bei bestimmten Vorsorgeuntersuchungen im Einzelfall der potenzielle Schaden den Nutzen überwiegt. Auch diese Entscheidung kann nur der behandelnde Arzt treffen, der die Patienten besser kennt, als sich dies über die GKV-Routinedaten ablesen ließe.

Schließlich sei erwähnt, dass der Erfolg der Prädiktionsmodelle insbesondere bei offenen Machine-Learning-Prozeduren zur Ermittlung kritischer Patienten(klassen) mit Handlungsbedarf im Wesentlichen von der IT-Infrastruktur und den Rechen- und Speicherkapazitäten der jeweiligen Krankenkassen abhängt. Dies könnte insbesondere bei kleineren Kassen zu Wettbewerbsnachteilen führen. Ebenso ist nicht ganz klar, ob eine Ansprache des Versicherten durch die Krankenkassen zwecks Bewerbung von Präventionsmaßnahmen mit Bonusprogrammen verknüpft werden darf, da die Ansprache der Krankenkassen selber nicht als eigentliche Präventionsleistung im Sinne der Bonusprogrammregelung § 65 SGBV angesehen werden könnte. Hierbei geht es nicht darum, mHealth-Präventionsmaßnahmen intern refinanzieren zu können, sondern darum, dem Versicherten einen Anreiz zu geben, von seiner Versicherung auch wirksam angesprochen und auf Präventionsangebote aufmerksam gemacht zu werden. Allerdings ist über das Präventionsgesetz die Möglichkeit gegeben über die Satzung der Krankenkassen Leistungen zur Prävention aufzunehmen. Eine Ansprache in Form personlisierter Informationskampagnen oder Werbung für Präventionsmaßnahmen könnte dann als verhaltenspräventive Maßnahme der Krankenkassen gelten.

Ein wichtiges Problem ist schließlich die verzögerte Übermittlung von Routinedaten durch die Leistungserbringer an die Krankenkassen. Dies macht eine in Echtzeit gesteuerte Informationsgenerierung für Präventionsempfehlungen unmöglich und schränkt wahrscheinlich den Anwendungsspielraum der hier vorgestellten Idee solange ein, bis dieses Problem behoben ist. Welche sinnvollen Empfehlungen mit den vorhandenen Daten bei einer deutlichen zeitlichen Verzögerung der Datenlieferung noch möglich sind, bleibt einem entsprechenden gesundheitsökonomischen Assessment zur Klärung vorbehalten.

## Fazit für die Praxis

- Das deutsche Gesundheitswesen muss sich zur finanziellen Konsolidierung und nachhaltigen Kostenkontrolle auf effektive Präventionsmaßnahmen refokussieren.
- Dieser Beitrag hat eine Idee vorgestellt, das Kostensenkungspotenzial durch aus Routinedatenanalysen abgeleiteten Präventionsmaßnahmen in Kombination mit effektivem Gesundheitsmarketing der Krankenkassen zu realisieren.
- GKV-Routinedaten (Gesetzliche Krankenversicherung) beinhalten die richtigen Ansatzpunkte zur unterstützenden beratenden Begleitung von Versicherten durch die Gesetzlichen Krankenkassen.
- Mittels kosteneffektiver Ansprache (z. B. via mHealth-Apps) sollte es gelingen, personalisierte Vorschläge für Präventionsmaßnahmen wirkungsvoll an Versicherte zu kommunizieren und schließlich umzusetzen.
- Informationen und Empfehlungen der Krankenkassen zu Präventionsmaßnahmen sind immer mit einem Arzt abzuklären.
- Die Implementation dieses Ansatzes könnte einen wichtigen Vorteil im Wettbewerb der Krankenkassen darstellen.

## Footnotes

1 Vgl. etwa S3-Leitlinie zum Prostatakarzinom im Internet unter https://www.leitlinienprogramm-onkologie.de/fileadmin/user_upload/Downloads/Leitlinien/Prostatatkarzinom/Version_6/LL_Prostatakarzinom_Langversion_6.2.pdf abgerufen am 01.09.2022.

2 Im § 1 SGB V:heißt es: „Die Krankenversicherung als Solidargemeinschaft hat die Aufgabe, die Gesundheit der Versicherten zu erhalten, wiederherzustellen oder ihren Gesundheitszustand zu bessern. Das umfasst auch die Förderung der gesundheitlichen Eigenkompetenz und Eigenverantwortung der Versicherten. … Die Krankenkassen haben den Versicherten dabei durch Aufklärung, Beratung und Leistungen zu helfen und unter Berücksichtigung von geschlechts-, alters- und behinderungsspezifischen Besonderheiten auf gesunde Lebensverhältnisse hinzuwirken.“.

3 Vgl. hierzu Pressemeldung des Bundessozialgerichtes vom 08.10.2019 im Internet unter https://www.bsg.bund.de/SharedDocs/Pressemitteilungen/DE/2019/2019_49.html. abgerufen am 21.11.2022.
